# Multi-Strain Tropical *Bacillus* spp. as a Potential Probiotic Biocontrol Agent for Large-Scale Enhancement of Mariculture Water Quality

**DOI:** 10.3389/fmicb.2021.699378

**Published:** 2021-08-11

**Authors:** Wei Ren, Haiwu Wu, Cong Guo, Bingqing Xue, Hao Long, Xiang Zhang, Xiaoni Cai, Aiyou Huang, Zhenyu Xie

**Affiliations:** ^1^State Key Laboratory of Marine Resource Utilization in the South China Sea, Hainan University, Haikou, China; ^2^Hainan Provincial Key Laboratory for Tropical Hydrobiology and Biotechnology, Hainan University, Haikou, China; ^3^College of Marine Sciences, Hainan University, Haikou, China; ^4^Laboratory of Development and Utilization of Marine Microbial Resource, Hainan University, Haikou, China

**Keywords:** multi-strain tropical *Bacillus* spp., probiotic biocontrol agent, marine recirculating aquaculture system, mariculture water quality, aquaculture wastewater

## Abstract

Aquaculture is suffering from long-term water eutrophication in intensive models, whereas the knowledge of multi-strain/specie for improving water quality is extremely limited. Herein, we aimed to develop multi-strain tropical *Bacillus* spp. as a potential probiotic biocontrol agent for large-scale enhancement of mariculture water quality. Given the practical application, the optimum multi-strain tropical *Bacillus* spp. (*B. flexus* QG-3, *B. flexus* NS-4, and *B. licheniformis* XCG-6 with the proportion 5: 5: 4) as a probiotic biocontrol agent was screened and obtained, which effectively improved water quality by removing chemical oxygen demand (COD), ammonia-nitrogen, and nitrate and significantly inhibited *Vibrio* spp. even at relatively low bacterial concentrations (10^4^ CFU/ml) in artificial feed wastewater and large-scale shrimp aquaculture ponds. More importantly, we found that the initial proportion of these three *Bacillus* sp. strains of multi-strain tropical *Bacillus* spp. markedly affected the final purification effects, whereas the initial concentration of that only influenced the purification rates at the early stage (0–48 h) instead of final purification effects. We reason that this multi-strain tropical *Bacillus* spp. as a good probiotic biocontrol agent could perform multiple actions, such as COD-degrading, nitrifying, denitrifying, and antagonistic actions, for large-scale enhancement of tropical aquaculture water. Additionally, the multi-strain tropical *Bacillus* spp. was safe for shrimp and could be stored for at least 240 days in spore form at room temperature. This multi-strain probiotic biocontrol agent may facilitate its adoption for further marine recirculating aquaculture system development and large-scale commercial application.

## Graphical Abstract

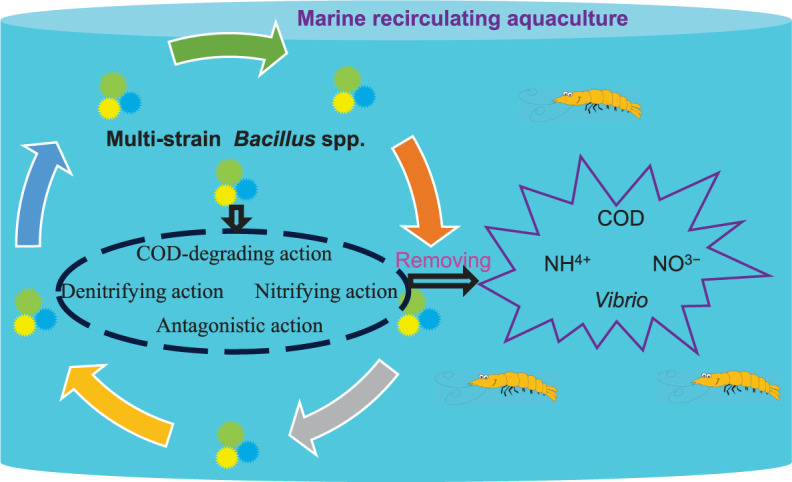

## Introduction

Generating/recycling water/resources from wastewater instead of just treating wastewater has become one of the most popular worldwide trends (Lu et al., 2019). Aquaculture contributed to about 44% of total fish production which is well-known as an important economic activity (Hamza et al., 2017), whereas wastewater from aquaculture is loaded with excessive organic matter, ammonia-nitrogen, and phosphorus, thereby causing a series of economic loss, establishment of antibiotic-resistant bacteria, and environmental problems, such as water eutrophication and atmospheric pollution due to the volatilization of ammonia and hydrogen sulfide (Lin and Chen, 2001, 2003; Kuhn et al., 2010; Hai, 2015; Guo et al., 2016; Li et al., 2020; Mariane De Morais et al., 2020; Wei et al., 2021; Xu et al., 2021). Reportedly, the resistance of harmful pathogenic microorganisms in aquaculture was caused by the abuse of antibiotics and chemicals, and the drug residues from aquatic products posed serious threats to human health through stepwise enrichment of food chains (Vaseeharan and Ramasamy, 2003; Hai, 2015; Paopradit et al., 2021; Wei et al., 2021). Therefore, exploring a harmless and recyclable biotechnology for wastewater treatment is necessary to ensure the sustainable development of aquaculture and for environmental protection.

Furthermore, with the enhancement of awareness of quality and safety of aquatic products, the growing concern for antibiotic-resistant microorganisms has led to strategies of alternative disease prevention methods, such as the application of non-pathogenic bacteria as potential alternative biocontrol agents (Vaseeharan and Ramasamy, 2003; Dash et al., 2017; Wei et al., 2021). Microorganisms such as potential probiotic biocontrol candidates based on the principle of competitive exclusion and immune stimulants play critical roles in preventing diseases, improving water quality, increasing the quantity and quality of aquaculture animals, and serving as potential food sources for aquatic animals (Moriarty, 1997; Blancheton et al., 2013; Dash et al., 2017; Hucheng et al., 2020; Wei et al., 2021). Although many probiotic biocontrol candidates offer a promising alternative to chemical and antibiotics in aquaculture, a process of screening and optimizing probiotic biocontrol candidates for particular aquatic animals plays an important role to make them species specific (Verschuere et al., 2000; Sun et al., 2010; Sadat Hoseini Madani et al., 2018; Wei et al., 2021). Additionally, probiotic biocontrol candidates can perform well in favorable conditions created by appropriate administration methods, which have been worldwide used through water routine or feed additives with either single or a combination of probiotics or even a mixture with prebiotics (Van Hai et al., 2007), thereby promoting enhancement of organic matter decomposition, reduction of nitrogen and phosphorus concentrations, and balance of ammonia, nitrite, and hydrogen sulfide (Boyd and Massaut, 1999; Ma et al., 2009; Cha et al., 2013).

*Bacillus* genera are commonly used in aquaculture, which can replace antibiotics and chemicals to prevent aquatic animal diseases and have no side effects on the aquaculture environment, thereby becoming a research hotspot of the processing of wastewater purification (Kuebutornye et al., 2019). Meanwhile, *Bacillus* genera were characterized by being rarely pathogenic and fast-growing and secreting high amounts of proteins, which were attractive species for the industry (Shah et al., 2016; Wei et al., 2021). *Bacillus* strains have been commonly selected as probiotics for humankind, applied in the aquaculture industry, mitigated nitrogen and phosphate pollution and a number of pathogenic bacteria, and especially associated with improving water quality by reducing organic matter accumulation (Rengpipat et al., 1998; Verschuere et al., 2000; Hai, 2015; Kumari et al., 2016; Saravanan et al., 2018; Sonune and Garode, 2018; Yi et al., 2018; Wang et al., 2019). Generally, *Bacillus* spp. are Gram-positive bacteria, which are better converters of organic matter back to CO_2_ than that of Gram-negative bacteria, thereby minimizing the buildup of dissolved and particulate organic carbon (Kuebutornye et al., 2019). However, most studies on *Bacillus* spp. focused on the use of single culture (Zokaeifar et al., 2014; Barman et al., 2018; De et al., 2018), while it is largely speculative whether two or even multiple combinations of strains/species would be beneficial. Reportedly, multi-strain/specie probiotics also enhanced protection against pathogenic infection (Timmerman et al., 2004; Kesarcodi-Watson et al., 2012).

We reason that *Bacillus* spp. based on a single strain are less effective than those based on multi-strain/specie probiotics in improving water quality. Therefore, the current research aimed to develop multi-strain tropical *Bacillus* spp. as a potential probiotic biocontrol agent for large-scale enhancement of mariculture water quality in shrimp aquaculture. The selected probiotic *Bacillus* spp. (Supplementary Figures 1, 2 and Supplementary Tables 1, 2) from Hainan tropical mariculture systems stored at the State Key Laboratory of Marine Resource Utilization in the South China Sea exhibited strong potential probiotic candidates, since they displayed high activities of protease and lipase and effectively removed chemical oxygen demand (COD) and nitrogen. The optimum multi-strain tropical *Bacillus* spp. as a probiotic biocontrol agent was obtained through gradual optimization and screening in this work, which can effectively improve mariculture water quality. These results will provide useful and practical strategies for the marine recirculating aquaculture system to ensure the sustainable development of aquaculture and for environmental protection.

## Materials and Methods

### Microorganisms

All tropical *Bacillus* strains used in this study, *B. licheniformis* XCG-1 and *B. flexus* NS-2 with COD removal efficiency, *B. flexus* QG-3 and *B. flexus* NS-4 with high lipase activity, *B. licheniformis* XCG-5 and *B. licheniformis* XCG-6 with high protease activity, and *B. flexus* XCG-7 and *B. flexus* XCG-8 with high removal ratio of nitrogen, were isolated from Hainan tropical mariculture systems and stored in the State Key Laboratory of Marine Resource Utilization in the South China. The eight *Bacillus* strains were identified by morphological observation (Supplementary Figure 1), phylogenetic analysis (Supplementary Figure 2), and physiological biochemical character (Supplementary Tables 1, 2), of which *B. flexus* QG-3, *B. flexus* NS-4, and *B. licheniformis* XCG-6 were registered in GenBank with accession numbers MZ404488, MZ404489, and MZ404490, respectively. Additionally, the commercial probiotic biocontrol product (containing *Bacillus subtilis*, *Lactobacillus acidilactici*, photosynthetic bacteria, Thiobacillus, denitrifying bacteria, *Actinomycetes*, nitrogen-releasing compounds, bio-enzymes, microelements, growth promoting factor, etc.; concentration of viable bacteria ≥ 10^9^ CFU/g) used as positive control were purchased from Hainan Zhengqiang Biochemical Technology Development Co., Ltd., Hainan, China.

### Preparation of Artificial Feed Wastewater

Artificial feed wastewater was as follows: 20 g/l artificial diet (containing 2 g/l eel powder and 0.4 g/l kelp powder), 10 ml/l skimmed milk, and 10 ml/l olive oil emulsion. The initial concentrations of COD, ammonia-nitrogen, nitrate, and pH in feed wastewater were adjusted to 720 mg/l, 76 mg/l, 62 mg/l, and 7.0 by artificial diet, (NH_4_)_2_SO_4_, NaNO_3_, and NaOH, respectively. Then, the feed wastewater was filtered by a 0.22-μm-pore-size filter to remove bacteria for further use. Artificial feed wastewater was used to optimize and screen the optimum multi-strain tropical *Bacillus* spp.

### Aquaculture Water and Breeding Management

Aquaculture water from five late-stage *Litopenaeus vannamei* ponds was used to confirm the ability of enhancement of mariculture water quality by the optimum multi-strain tropical *Bacillus* spp. The experimental ponds and aquaculture management are shown in Table 1.

**TABLE 1:** Late-stage *L. vannamei* ponds and aquaculture management.

	Late-stage *L. vannamei* ponds				
	1^#^	2^#^	3^#^	4^#^	5^#^
Size (m^2^)	2,800	3,133	3,000	2,600	2,533
Water depth (m)	1.8	1.8	1.9	1.6	1.8
Healthy condition of *L. vannamei*	Unhealthy	Unhealthy	Unhealthy	Healthy	Healthy
*Vibrio* (CFU/ml)	400	500	470	150	160
COD (mg/l)	72	121.5	73.5	42	20.5
DO (mg/l)	6.28	5.15	5.1	5.75	6.38
Ammonia-nitrogen (mg/l)	2.1	3.15	2.98	1.12	1.08
Nitrites (mg/l)	0.01	0.05	0.05	0.005	0.005
Nitrates (mg/l)	0.95	1.75	1.72	0.5	0.5
Water exchange (t/d)	0	0	0	0	0
Feed intake (kg/d)	6	6	6	6	6
Other probiotics	No	No	Yes	No	No
Treatment	Multi-strain *Bacillus* spp.	Multi-strain *Bacillus* spp. + 30% sugarcane molasses	Commercial probiotic biocontrol product	30% sugarcane molasses	Control

*Late-stage L. vannamei ponds with the treatment of commercial probiotic biocontrol product (3^#^ pond) and without adding multi-strain tropical Bacillus spp. or 30% sugarcane molasses (5^#^ pond) were as positive control and negative control, respectively. The initial concentrations of multi-strain tropical Bacillus spp. (B. flexus QG-3, B. flexus NS-4, and B. licheniformis XCG-6 with the proportion 5: 5: 4) and commercial probiotic biocontrol product in the treatment aquaculture ponds were adjusted to 10^4^ CFU/ml.*

### Plackett–Burman Design

Plackett-Burman design, an effective method for screening significant factors, involves a large number of factors affecting the process and relatively few runs (Asfaram et al., 2016; Venkataraghavan et al., 2020). In this part of the study, Plackett–Burman design was carried out to investigate the effect degrees of independent variables (*B. licheniformis* XCG-1, *B. flexus* NS-2, *B. flexus* QG-3, *B. flexus* NS-4, *B. licheniformis* XCG-5, *B. licheniformis* XCG-6, *B. flexus* XCG-7, and *B. flexus* XCG-8) on COD removal efficiency in the process of artificial feed wastewater purification, and to screen some important *Bacillus* spp. strains for further optimization. Twelve groups of experiments with triplicates for each group were designed with eight *Bacillus* spp. strains (*B. licheniformis* XCG-1, *B. flexus* NS-2, *B. flexus* QG-3, *B. flexus* NS-4, *B. licheniformis* XCG-5, *B. licheniformis* XCG-6, *B. flexus* XCG-7, and *B. flexus* XCG-8) by 12-factor-2-level Plackett–Burman design for selecting the significant parameters. The experimental design and results used in this study are shown in Table 2. The COD removal efficiency (%) after treating for 24 h was taken as the dependent or response. COD was detected by a Hach DR/2400 spectrophotometer (HACH^®^ Company, Loveland, CO., United States).

**TABLE 2:** Twelve runs with 8 parameters in Plackett–Burman design.

Run #	Factor								COD removal efficiency (%)
	X1	X2	X3	X4	X5	X6	X7	X8	
1	1	−1	−1	−1	1	−1	1	1	40.58 ± 1.42
2	−1	−1	−1	1	−1	1	1	−1	46.11 ± 0.69
3	−1	1	−1	1	1	−1	1	1	40.08 ± 1.48
4	−1	−1	1	−1	1	1	−1	1	58.82 ± 1.50
5	1	1	−1	−1	−1	1	−1	1	50.14 ± 1.63
6	−1	1	1	1	−1	−1	−1	1	61.96 ± 2.33
7	1	−1	1	1	1	−1	−1	−1	59.91 ± 0.97
8	1	−1	1	1	−1	1	1	1	69.62 ± 1.66
9	−1	−1	−1	−1	−1	−1	−1	−1	22.22 ± 1.59
10	1	1	1	−1	−1	−1	1	−1	55.79 ± 0.96
11	1	1	−1	1	1	1	−1	−1	53.79 ± 1.72
12	−1	1	1	−1	1	1	1	−1	59.91 ± 1.36

*eight parameters (Bacillus spp. strains) were shown as code levels. X1, B. licheniformis XCG-1; X2, B. flexus NS-2; X3, B. flexus QG-3; X4, B. flexus NS-4; X5, B. licheniformis XCG-5; X6, B. licheniformis XCG-6; X7, B. flexus XCG-7; X8, B. flexus XCG-8. The selections of low (−1) and high (1) levels for each factor represent that the concentration of a single strain was set at 2 × 10^5^ CFU/ml and 0 CFU/ml, respectively, which was based on the results of pre-experiment.*

### The Path of Steepest Ascent

The path of steepest ascent was applied to approach the optimal level of the significant variables. Actually, the concentration of each main effect factor (*Bacillus* sp.) obtained from the Plackett–Burman design was far from the actual optimum. The significant factors should be roughly optimized to determine the center point of each variable for the next Box–Behnken design (Vi et al., 2017). Therefore, six runs with the response as the COD removal efficiency of feed wastewater were evaluated with triplicates for each run by the path of steepest ascent after treating for 24 h (Table 3).

**TABLE 3:** Experimental design and results of the path of steepest ascent.

Run #	Factor (× 10^5^ CFU/ml)			COD removal efficiency (%)
	X3	X4	X6	
1	2.0	2.0	2.0	63.50 ± 1.48
2	3.0	2.7	2.6	64.94 ± 0.92
3	4.0	3.4	3.2	70.88 ± 2.06
4	5.0	4.1	3.8	75.13 ± 1.38
5	6.0	4.8	4.4	72.50 ± 0.88
6	7.0	5.5	5.0	71.19 ± 0.75

*X3, B. flexus QG-3; X4, B. flexus NS-4; X6, B. licheniformis XCG-6.*

### Box–Behnken Design

Using the path of steepest ascent design, the significant factors were roughly optimized to determine the center points of each variable for Box–Behnken design. Box–Behnken was employed to provide the optimum levels of the three variables (X3, *B. flexus* QG-3; X4, *B. flexus* NS-4; X6, *B. licheniformis* XCG-6). Fifteen runs with three replications at the center points were conducted to determine the relationship of variance and approach the maximum response (COD removal efficiency at 24 h after treatment; Table 4). The regression and graphical analyses of the experimental data were analyzed and generated by the “Design Expert” statistical package (Design-Expert Software Version 12.0.3.0, Stat-Ease Inc., Minneapolis, MN, United States). The quality of the fit of quadratic model was expressed by the coefficient of determination (*R*^2^), and its statistical significance was checked by the *F*-test. The behavior of the system is explained by the following quadratic equation:

$$Y=\beta_{0}+\sum_{i=1}^{8}\beta_{\text{i}}X_{\text{i}}+\sum_{i=1}^{8}\beta_{\text{ii}}X_{\text{i}}^{2}+\sum_{i=1}^{7}\sum_{j=i+1}^{8}\beta_{\text{ij}}X_{\text{i}}X_{\text{j}}+\varepsilon .$$

**TABLE 4:** Fifteen runs with three parameters in Box–Behnken design.

Run #	Factor (× 10^5^ CFU/ml)			COD removal efficiency (%)
	X3	X4	X6	
1	5	3.4	3.2	71.04 ± 1.26
2	5	3.4	4.4	72.92 ± 0.89
3	5	4.8	3.2	77.17 ± 1.31
4	5	4.8	4.4	78.05 ± 1.05
5	4	4.1	3.2	70.80 ± 2.04
6	6	4.1	3.2	74.58 ± 0.78
7	4	4.1	4.4	71.67 ± 0.86
8	6	4.1	4.4	79.96 ± 1.74
9	4	3.4	3.8	71.67 ± 2.45
10	4	4.8	3.8	83.21 ± 1.52
11	5	3.4	3.8	78.33 ± 1.42
12	5	4.8	3.8	83.00 ± 1.32
13	4	4.1	3.8	83.08 ± 1.05
14	4	4.1	3.8	82.25 ± 1.62
15	4	4.1	3.8	82.96 ± 1.35

*X3, B. flexus QG-3; X4, B. flexus NS-4; X6, B. licheniformis XCG-6. Y is the predicted response [COD removal efficiency (%)], β_0_ is the intercept, β_i_ is the linear coefficient, X_i_ is the factor variable coded, β_ii_ is the quadratic coefficient for the factor, β_ij_ is the interaction effect, and ε is the error (Muhamad et al., 2013; Ren et al., 2016). The significance of all terms in the polynomial was judged statistically according to the p-value which was compared with the significance level of 0.05.*

### Verification

#### Large-Scale Production of Multi-Strain Tropical *Bacillus* spp.

*Bacillus* spp. culture medium developed by our lab (containing 10 g/l sugarcane molasses, 8 g/l rice bran, 2 g/l corn flour, 2 g/l K_2_HPO_4_, 0.05 g/l MnSO_4_⋅H_2_O, and 0.1 g/l MgSO_4_⋅7H_2_O; pH 7.0) was used for large-scale fermentation of *B. flexus* QG-3, *B. flexus* NS-4, and *B. licheniformis* XCG-6 in a 500-l stirred-tank fermenter (fermentation conditions: aeration rate 1.5 vvm, tank pressure 0.03–0.05 MPa, agitation speed 90 rpm, temperature 30°C), respectively. After individual fermentation for 36 h, the fermentation broth of *B. flexus* QG-3, *B. flexus* NS-4, and *B. licheniformis* XCG-6 were separately put into cans for further use (Supplementary Figure 3). The activities of proteinase and lipase in fermentation broth were detected using fat-free milk (Nygren et al., 2007) and rhodamine B (El Aamri et al., 2020), respectively. Bacterial suspension with the appropriate dilution gradient of multi-strain tropical *Bacillus* spp. obtained by large-scale fermentation was plated out on the *Bacillus* spp. culture medium agar plates and incubated overnight at 30°C, and hence the total number of viable *Bacillus* spp. as colony-forming units (CFU)/ml existed in fermentation as CFU was obtained. Additionally, the evaluation of storage stability of mixed fermentation broth (5.17 × 10^5^ CFU/ml *B. flexus* QG-3, 4.79 × 10^5^ CFU/ml *B. flexus* NS-4, and 3.85 × 10^5^ CFU/ml *B. licheniformis* XCG-6) was performed at room temperature for 240 days.

#### Purification Effects of Multi-Strain Tropical *Bacillus* spp. on Artificial Feed Wastewater

The optimum multi-strain tropical *Bacillus* spp. was 5.17 × 10^5^ CFU/ml *B. flexus* QG-3, 4.79 × 10^5^ CFU/ml *B. flexus* NS-4, and 3.85 × 10^5^ CFU/ml *B. licheniformis* XCG-6 according to the results of optimization, indicating that *B. flexus* QG-3, *B. flexus* NS-4, and *B. licheniformis* XCG-6 in the proportion 5.17: 4.79: 3.85 (in theory) at the 10^5^ CFU/ml level as a potential probiotic biocontrol agent can effectively enhance the aquaculture wastewater quality. Given the practical application, the initial concentrations of multi-strain tropical *Bacillus* spp. (*B. flexus* QG-3, *B. flexus* NS-4, and *B. licheniformis* XCG-6 with the proportion 5: 5: 4) in 10 l artificial feed wastewater were adjusted to 10^4^ CFU/ml, 10^5^ CFU/ml, 10^6^ CFU/ml, 10^7^ CFU/ml, and 10^8^ CFU/ml. Artificial feed wastewater without adding multi-strain tropical *Bacillus* spp. and that with commercial probiotic biocontrol product at the 10^8^ CFU/ml level were as negative control group and positive control group, respectively.

#### Purification Effects of Multi-Strain Tropical *Bacillus* spp. on Mariculture Water

Five late-stage *L. vannamei* ponds (Table 1) with the treatments of multi-strain *Bacillus* spp., multi-strain *Bacillus* spp. + 30% sugarcane molasses, 30% sugarcane molasses, commercial probiotic biocontrol product, and control without adding anything were used to confirm the ability of optimum multi-strain tropical *Bacillus* spp. to improve mariculture water quality. Given the practical application, the initial concentrations of multi-strain tropical *Bacillus* spp. (*B. flexus* QG-3, *B. flexus* NS-4, and *B. licheniformis* XCG-6 with the proportion 5: 5: 4) and commercial probiotic biocontrol product in aquaculture ponds were adjusted to 10^4^ CFU/ml. The concentrations of COD, ammonia-nitrite, and nitrate, as well as pH and the inhibition of *Vibrio* spp. in aquaculture ponds, were continuously detected during this experiment. Three parallel site samples with a uniform distribution from each pond were collected within 1 m of the shore at a depth of approximately 30 cm from the water surface.

For each sample, pH and DO (dissolved oxygen) were measured by a pH meter (AB15, Fisher Scientific, Waltham, MA, United States) and a DO tester (Oxi 3205SET3, WTW, Germany), respectively. COD, ammonia-nitrite, and nitrate were analyzed using a Hach DR/2400 spectrophotometer (HACH^®^ Company, Loveland, CO., United States). For the inhibition of *Vibrio* spp. detection, the inhibition ratio of the different treatments was calculated through the following equation:

Inhibitionratio(%)=[C-T]/C×100%

where *C* is the initial number of *Vibrio* spp. (CFU/ml) in each pond as the control; *T* is the number of *Vibrio* spp. (CFU/ml) at different periods after the treatments of multi-strain *Bacillus* spp., multi-strain *Bacillus* spp. + 30% sugarcane molasses, 30% sugarcane molasses, commercial probiotic biocontrol product, and the control without adding anything.

## Results

### Screening of *Bacillus* spp. Strains for Enhancement of Aquaculture Wastewater by Plackett–Burman Design

**FIGURE 1:**
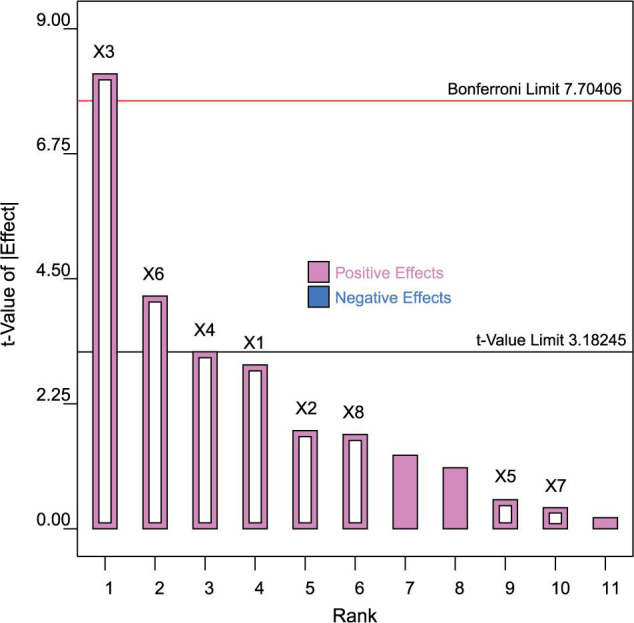
Pareto chart showing the effect of *Bacillus* spp. strains. X1, *B. licheniformis* XCG-1; X2, *B. flexus* NS-2; X3, *B. flexus* QG-3; X4, *B. flexus* NS-4; X5, *B. licheniformis* XCG-5; X6, *B. licheniformis* XCG-6; X7, *B. flexus* XCG-7; X8, *B. flexus* XCG-8.

The experiment design matrix with *Y* (COD removal efficiency) as responses is listed in Table 1, and the results and analysis of variance (ANOVA) are presented in Table 5. After fitting a first-order polynomial model, the coefficient of determination (*R*^2^) was about 0.97, which indicated a good fit. For the COD removal efficiency, three independent variables (X3, *B. flexus* QG-3; X4, *B. flexus* NS-4; X6, *B. licheniformis* XCG-6) presented significantly positive effects (Table 5). Generally, the variable with a confidence level above 95% (*p* < 0.05) is considered as a significant parameter. These results were confirmed from the Pareto chart as shown in Figure 1, indicating that higher effects presented in the upper portion and then progressed down to the lower effects. In addition, the “Adequacy precision” value of 13.73 for COD removal efficiency, greater than 4, demonstrated that the signal was adequate. Therefore, taking the COD removal efficiency, *B. flexus* QG-3, *B. flexus* NS-4, and *B. licheniformis* XCG-6 were selected as the important bacteria for further concentration optimization.

**TABLE 5:** ANOVA for 12-run Plackett–Burman design and model fitting.

Source	Sum of squares	df	Mean square	*F-*value	*P*-value (Prob > *F*)	
Model	1746.5500	8	218.3187	13.7325	0.0271	Significant
X1	138.2444	1	138.2444	8.6957	0.0601	
X2	49.65401	1	49.6540	3.1233	0.1753	
X3	1065.7790	1	1065.7790	67.0388	0.0038	Significant
X4	161.4067	1	161.4067	10.1527	0.0499	Significant
X5	4.3802	1	4.3802	0.2755	0.6360	
X6	278.8852	1	278.8852	17.5422	0.0248	Significant
X7	2.2969	1	2.2969	0.1445	0.7292	
X8	45.9034	1	45.9034	2.8874	0.1878	
Residual	47.6938	3	15.8979			
Cor total	1794.2440	11				

**Credibility analysis of the regression equations**						

Index mark	SD	Mean	CV%	*R* ^2^	Adj. *R*^2^	Adequacy precision
COD removal efficiency (%)	3.99	51.58	7.73	0.97	0.90	13.73

*X1, B. licheniformis XCG-1; X2, B. flexus NS-2; X3, B. flexus QG-3; X4, B. flexus NS-4; X5, B. licheniformis XCG-5; X6, B. licheniformis XCG-6; X7, B. flexus XCG-7; X8, B. flexus XCG-8.*

### Optimizing of Bacillus spp. Strains for Enhancement of Aquaculture Wastewater by Path of Steepest Ascent and Box–Behnken Design

Based on the results of Plackett–Burman design, the most significant factors (*B. flexus* QG-3, *B. flexus* NS-4, and *B. licheniformis* XCG-6) were conducted in the path of steepest ascent. The experiment design and responses for the path of steepest ascent design are shown in Table 3. The highest COD removal efficiency (75.13%) was reported at run 4 with 5 × 10^5^ CFU/ml *B. flexus* QG-3, 4.1 × 10^5^ CFU/ml *B. flexus* NS-4, and 3.8 × 10^5^ CFU/ml *B. licheniformis* XCG-6. The levels of three factors at run 4 were used in the optimization step through Box–Behnken design.

Box–Behnken design was employed to optimize the key factors (X3, *B. flexus* QG-3; X4, *B. flexus* NS-4; X6, *B. licheniformis* XCG-6) and evaluate their interactions on the COD removal efficiency in the process of artificial feed wastewater purification. The combination of independent variables, results, and ANOVA are listed in Tables 4, 6. The statistical significance of each coefficient of regression equation was checked by Fisher’s “*F* statistics” value (*F*-value) and probability value (*p*-value), thus in turn indicating the interactions of the variables. Generally, the model with a very small *p*-value (*p* < 0.05) indicates that the model term is significant; meanwhile, the larger *F*-value and lower *P*-value indicate the significance of each term. From Table 6, the coefficients of linear term (X3, X4) and quadratic term (X3^2^ and X6^2^) were significant for COD removal efficiency. The results listed in Table 6 indicated that the models were significant and adequate for reasonable prediction of COD removal efficiency, within the variable range employed, as evidenced by the *F*-value (20.4212) and the low probability values (0.002). It also indicated that the chance for model *F*-values of this size occurred because of the statistical noise which was only 0.2%. The “Lack of fit” test was used to measure the failure of the model to fit the experiment data. The “Lack of fit” gave *F*-values for COD removal efficiency of 14.1351 and *p*-values for the response of 0.0668. The results suggested that the “Lack of fit” was non-significant relative to the pure error, with probabilities of 6.68% for occurrence for COD removal efficiency of this large value due to noise. The result indicated that the models could fit the experimental values and excellently predict COD removal efficiency.

**TABLE 6:** ANOVA for 15-run Box–Behnken design and model fitting.

Source	Sum of squares	df	Mean square	*F*-value	*P*-value (Prob > *F*)	
Model	328.4645	9	36.4961	20.4212	0.0020	Significant
X3	42.8738	1	42.8738	23.9898	0.0045	Significant
X4	94.3251	1	94.3251	52.7791	0.0008	Significant
X6	10.1475	1	10.1475	5.6780	0.0629	
X3 X4	11.7992	1	11.7992	6.6022	0.0501	
X3 X6	5.0850	1	5.0850	2.8453	0.1524	
X4 X6	0.2500	1	0.2500	0.1400	0.7237	
X3^2^	16.6992	1	16.6992	9.3440	0.0282	Significant
X4^2^	9.2662	1	9.2662	5.1848	0.0718	
X6^2^	150.4895	1	150.4895	84.2056	0.0003	Significant
Residual	8.9358	5	1.7872			
Lack of fit	8.5334	3	2.8445	14.1351	0.0668	Not significant
Pure error	0.4025	2	0.2012			
Cor total	337.4003	14				

**Credibility analysis of the regression equations**						

Index mark	SD	Mean	CV%	*R* ^2^	Adj. *R*^2^	Adequacy precision
COD removal efficiency (%)	1.34	77.38	1.73	0.97	0.93	12.00

*X3, B. flexus QG-3; X4, B. flexus NS-4; X6, B. licheniformis XCG-6.*

A higher coefficient of determination (*R*^2^) of the quadratic regression model indicates that the model is workable. Therefore, the *R*^2^ of COD removal efficiency was 0.97 and the adjusted *R*^2^ was 0.93 which were in reasonable agreement with *R*^2^, demonstrating a high degree of correlations between the experimental data and predicted values. In general, a low coefficient of variation indicates that the model is adequate and owns high precision and reliability for fitting experimental values. In this case, the coefficient of variances (CV) for COD removal efficiency was 1.73, which was low enough to represent the data adequately. In addition, the “Adequacy precision” value of 12 for COD removal efficiency, greater than 4, demonstrating that the signal was adequate. The empirical equation developed for COD removal efficiency is listed as follows:

Y=-343.51371+26.50048X3+45.94566X4+129.69858X6-2.45357X3X4+1.87917X3X6-0.59524X4X6-2.12667X3-23.23299X4-217.73380X62

**FIGURE 2:**
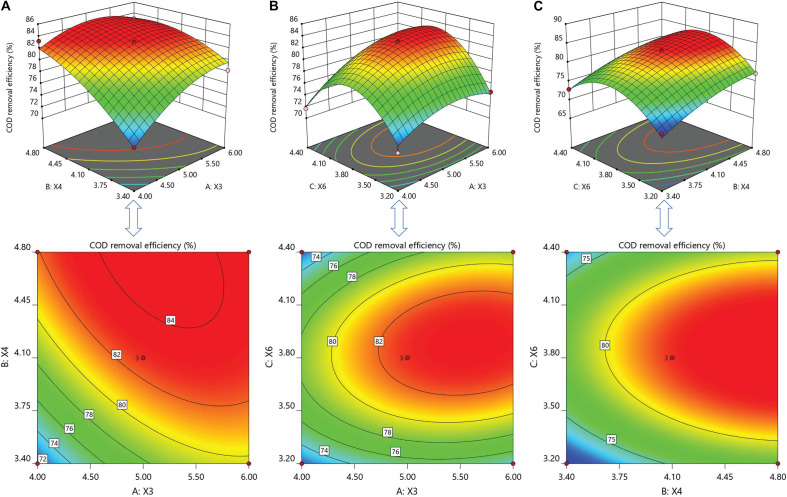
Response surface and contour plots for the effect of variables on COD removal efficiency. **(A)** For the effect of A: *B. flexus* QG-3 (X3) and B: *B. flexus* NS-4 (X4); **(B)** for the effect of A: *B. flexus* QG-3 (X3) and C: *B. licheniformis* XCG-6 (X6); **(C)** for the effect of B: *B. flexus* NS-4 (X4) and C: *B. licheniformis* XCG-6 (X6).

In order to study and visualize the influences of factors and their mutual interactions on COD removal efficiency, three-dimensional (3D) response surfaces and two-dimensional (2D) response contours were plotted by the response (*Z*-axis) according to two factors (*X* and *Y* coordinates), holding the other one factor at zero (0 level). The interactions between *B. flexus* QG-3 (X3) and *B. flexus* NS-4 (X4) are presented in Figure 2A, keeping the concentration of *B. licheniformis* XCG-6 at 0 level. For COD removal efficiency, *B. flexus* QG-3 (X3) and *flexus* NS-4 (X4) contributed significant influences in a linear manner, while the interactive effects between each other presented non-significant effects. As the concentrations of *B. flexus* QG-3 and *B. flexus* NS-4 increased, the COD removal efficiency increased significantly and then decreased slightly. Figure 2B presents the interaction of *B. flexus* QG-3 (X3) and *B. licheniformis* XCG-6 (X6), with a fixed *B. flexus* NS-4 (0 level). The linear and quadratic terms of *B. flexus* QG-3 caused significant influences on COD removal efficiency, while *B. licheniformis* XCG-6 only contributed significant influences in quadratic terms. Figure 2C shows the response surfaces of *B. flexus* NS-4 (X4) and *B. licheniformis* XCG-6 (X6), with a fixed *B. flexus* QG-3. The significant effects on COD removal efficiency were caused by the linear manner of *B. flexus* NS-4 and quadratic manner of *B. licheniformis* XCG-6, while the interactive effects *B. flexus* NS-4 and *B. licheniformis* XCG-6 produced non-significant influences on COD removal efficiency. All of the interactions of individual *Bacillus* sp. to each other presented non-significant effects (Table 6).

After optimization by Box–Behnken design, the predicted optimum multi-strain tropical *Bacillus* spp. was 5.17 × 10^5^ CFU/ml *B. flexus* QG-3, 4.79 × 10^5^ CFU/ml *B. flexus* NS-4, and 3.85 × 10^5^ CFU/ml *B. licheniformis* XCG-6, respectively. Using this optimum multi-strain tropical *Bacillus* spp., the theoretical COD removal efficiency was found to about 84.70%.

### Verification Test

To confirm the validity of the statistical experimental strategies for large-scale application of wastewater purification, firstly, *B. flexus* QG-3, *B. flexus* NS-4, and *B. licheniformis* XCG-6 were separately obtained by large-scale cultivation (Supplementary Figure 3). In turn, the optimum multi-strain tropical *Bacillus* spp. (*B. flexus* QG-3, *B. flexus* NS-4, and *B. licheniformis* XCG-6 in the proportion 5: 5: 4) and commercial probiotic biocontrol product (as the positive control) were used to treat artificial feed wastewater and aquaculture water of *L. vannamei* ponds.

**FIGURE 3:**
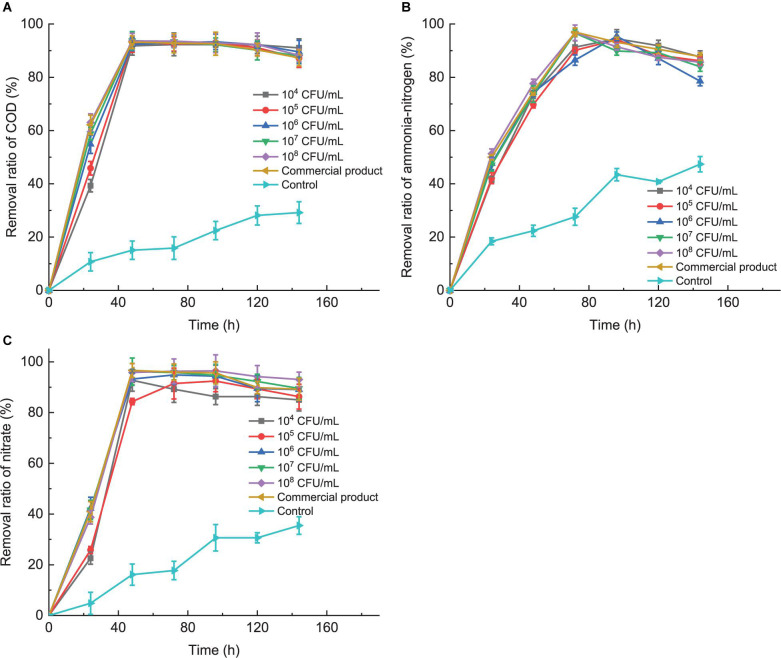
The purification effects of multi-strain tropical *Bacillus* spp. (*B. flexus* QG-3, *B. flexus* NS-4, and *B. licheniformis* XCG-6 in the proportion 5: 5: 4) on removal ratios of COD **(A)**, ammonia-nitrogen **(B)**, and nitrate **(C)** of artificial feed wastewater. The initial concentrations of multi-strain tropical *Bacillus* spp. in 10 l artificial feed wastewater were adjusted to 10^4^ CFU/ml, 10^5^ CFU/ml, 10^6^ CFU/ml, 10^7^ CFU/ml, and 10^8^ CFU/ml. Artificial feed wastewater without adding multi-strain tropical *Bacillus* spp. and with commercial probiotic biocontrol product at 10^8^ CFU/ml level were as negative control and positive control, respectively.

As shown in Figure 3A, COD removal ratios of artificial feed wastewater displayed the rapidly increasing rate in a 24-h period and then decreased slightly in a 24–48-h period. The COD removal ratio of artificial feed wastewater reached a maximum (above 90%) after treating with multi-strain tropical *Bacillus* spp. and commercial product in a 48-h period, while COD content did not change significantly in the 48–96-h period. We reason that the organic matter of artificial feed wastewater was sufficient for microorganisms as nutrients for their own growth in the 48-h period. After 96 h, with the depletion of organic matter in artificial feed wastewater, the dead bacteria and extracellular products led to COD content slightly increasing. Meanwhile, the trend of removal ratios of ammonia-nitrogen (Figure 3B) and nitrate (Figure 3C) was similar to that of COD in artificial feed wastewater, which indicated that these strains could not only effectively remove COD but also simultaneously remove ammonia-nitrogen and nitrate. The removal ratio of nitrate was up to the maximum in the 48-h period, while that of ammonia-nitrogen reached the maximum at 72 h. This delay may be due to that multi-strain tropical *Bacillus spp.* firstly promoted the organic matter decomposition, thereby resulting in accumulation of ammonia-nitrogen, and then conversion of ammonia-nitrogen to nitrite, whereas initial nitrate in wastewater was conversed immediately. Interestingly, the initial concentration of multi-strain tropical *Bacillus* spp. almost did not influence the final results of purification effects, which only had the effect on the purification rate of treatment in the early stage (0–48 h). However, the initial proportion of *B. flexus* QG-3, *B. flexus* NS-4, and *B. licheniformis* XCG-6 in multi-strain tropical *Bacillus* spp. compared with the initial concentration of multi-strain tropical *Bacillus* spp. played a key role in the purification effects according to the results of optimization (Figure 3).

**FIGURE 4:**
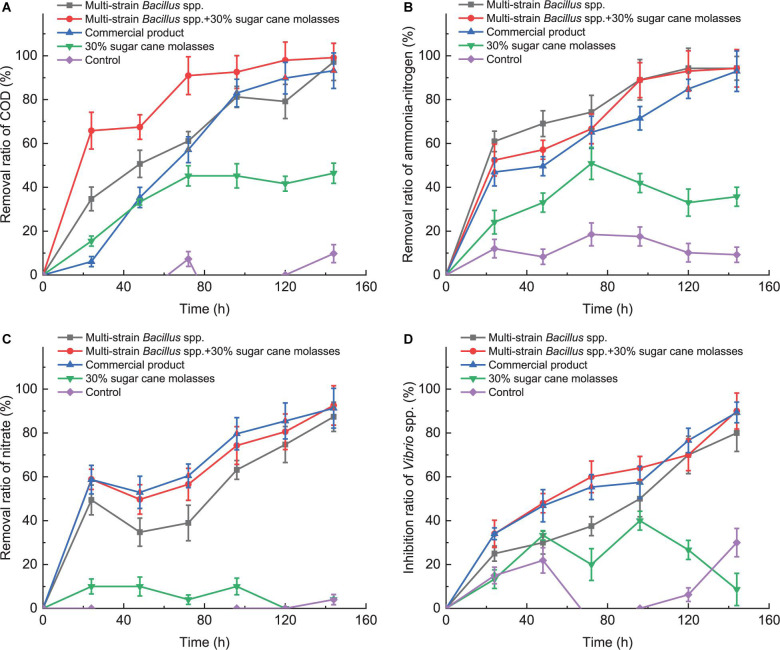
The purification effects of multi-strain tropical *Bacillus* spp., multi-strain *Bacillus* spp. + 30% sugarcane molasses, 30% sugarcane molasses, commercial probiotic biocontrol product, and control without adding anything on removal ratios of COD **(A)**, ammonia- nitrogen **(B)**, and nitrate **(C)**, and inhibition ratio of *Vibrio* spp. of aquaculture water of *L. vannamei* ponds **(D)**. The initial concentrations of multi-strain tropical *Bacillus* spp. (*B. flexus* QG-3, *B. flexus* NS-4, and *B. licheniformis* XCG-6 with the proportion 5: 5: 4) and commercial probiotic biocontrol product in aquaculture ponds were adjusted to 10^4^ CFU/ml.

As shown in Figures 4A–C, the removal ratios of COD, ammonia-nitrogen, and nitrate of aquaculture water of *L. vannamei* ponds displayed the rapidly increasing rate in the 24-h period and then decreased slightly in the 24–48-h period, which was similar to that of artificial feed wastewater, indicating that due to the rapid growth and reproduction of the strains, considerable amounts of nutrient salts and organic matter were used and degraded during this period. Meanwhile, DO of aquaculture water of *L. vannamei* ponds with the treatment of these bacteria maintained at a high level in the 24–48-h period (Supplementary Figure 4), indicating that these *Bacillus* spp. were aerobic bacteria with low biochemical oxygen demand and would not compete for oxygen with aquaculture animals. In Figure 4D, with the growth and reproduction of *Bacillus* spp., the number of *Vibrio* spp. in ponds decreased continuously, indicating that these *Bacillus* spp. can effectively inhibit the growth and reproduction of *Vibrio* spp. in *L. vannamei* ponds by competition nutrition. The sugarcane molasses accelerated the removal ratios of COD, nitrate, and *Vibrio* of multi-strain *Bacillus* spp., whereas they did not influence their final purification effects (Figures 4A,B,D). Additionally, non-significant mortality was observed in every pond during the testing period, indicating that multi-strain *Bacillus* spp. was harmless to shrimp.

### Storage Stability of Multi-Strain Tropical *Bacillus* spp.

The storage stability of multi-strain tropical *Bacillus* spp. was evaluated by monitoring the total number of viable bacteria, protease activity, and pH (Table 7). After 240 days, the total number of viable bacteria of multi-strain tropical *Bacillus* spp. was maintained above 10^8^ CFU/ml in spore form, pH decreased from 7.2 to 5.7, and protease activity was still maintained at a relatively high level, indicating that the multi-strain tropical *Bacillus* spp. can be stored for a long time at room temperature.

**TABLE 7:** Storage stability of multi-strain tropical *Bacillus* spp. at room temperature.

Time (d)	Viable bacteria (CFU/ml)	Protease activity (U/ml)	pH
0	× 10^9^	23.47 ± 0.67	7.2 ± 0.2
80	× 10^9^	17.74 ± 1.03	6.5 ± 0.3
160	× 10^8^	14.36 ± 0.71	5.7 ± 0.2
240	× 10^8^	10.28 ± 1.15	5.7 ± 0.3

## Discussion

Currently, biological treatment as a harmless and recyclable technology for aquaculture wastewater treatment provides an eco-friendly method where microorganisms were added at certain concentrations to ensure the sustainable development of aquaculture instead of depending on only the freshwater source (Wei et al., 2021). As we all know, beneficial bacteria called probiotics give a much more efficient approach to alternate chemotherapeutic agents, which are sustainable aquaculture as well as being environment-friendly (Nimrat et al., 2012). Foremost among probiotics, photosynthetic bacteria (Chang et al., 2019; Lu et al., 2019), yeast (Gao et al., 2019), *Lactobacillus* spp. (Dawood et al., 2020; Zheng et al., 2020), and *Pseudomonas* spp. (Ruan et al., 2020; Zhang et al., 2020), especially *Bacillus* spp. (Barman et al., 2018; Gao et al., 2018), have advantages to the enhancement of aquaculture water quality. *Bacillus* as a potential probiotic biocontrol agent has been commonly chosen to improve water quality for sustainable aquaculture by reducing organic matter, ammonia-nitrogen, and phosphorus accumulation and inhibiting certain pathogenic bacteria of fishery by producing antimicrobial peptides, such as *B. megaterium* (Luo et al., 2016; Gao et al., 2018), *B. cereus* (Lalloo et al., 2007; Lalloo et al., 2010), *B. subalis* (Lalloo et al., 2007), and *B. licheniformis* (Lalloo et al., 2007), *B. velezensis* (Thurlow et al., 2019), whereas most of these literatures focused on a single probiotic bacterium for wastewater purification. We reason that a good probiotic biocontrol agent should have the capability of effectively removing multi-waste compounds from wastewater. Given this point, a consortium of probiotic bacteria will be advantageous over a single culture for improving the removal ratio of multi-waste compounds due to the cooperative interactions between the cocultivated probiotic bacteria. To date, not much attempt has been made to focus on the aquaculture wastewater purification through microbial consortium. Meanwhile, the tropical marine region with unique climate and environmental condition harbors diverse probiotic bacteria with unique metabolic and physiological capabilities, and hence success is greatly dependent on having the right microbes with the capabilities in the right environments for the degradation process to occur (Ren et al., 2018; Ren et al., 2019a, b; Wei et al., 2021). Therefore, the aim of this work was to assemble a bacterial consortium as a potential probiotic biocontrol agent for large-scale enhancement of tropical mariculture water quality because of its more efficiency in the removal of multi-waste compounds in effluent water than single cultures.

In the long-term natural evolution, Hainan has formed a unique tropical microbial community structure, and there are abundant resources of *Bacillus* spp. that are highly adaptable to the tropical marine environment (Wei et al., 2021). Fortunately, based on the indigenous *Bacillus* spp. strains isolated from the tropical mariculture environment in Hainan, the optimum multi-strain *Bacillus* spp. in theory, 5.17 × 10^5^ CFU/ml *B. flexus* QG-3, 4.79 × 10^5^ CFU/ml *B. flexus* NS-4, and 3.85 × 10^5^ CFU/mL *B. licheniformis* XCG-6, as a potential probiotic agent, were established for artificial feed wastewater purification through optimization in this work.

Given the practical application, the theoretical *Bacillus* spp. (*B. flexus* QG-3, *B. flexus* NS-4, and *B. licheniformis* XCG-6) proportion of multi-strain tropical species were adjusted to 5: 5: 4, and artificial feed wastewater and aquaculture ponds were used to confirm their efficiency, which displayed high ability of improvement of mariculture water quality by removing COD, ammonia-nitrogen, and nitrate and significantly inhibited *Vibrio* spp. at relatively low bacterial concentrations (10^4^ CFU/ml). Moreover, we found that the initial proportion of *Bacillus* strains in multi-strain tropical *Bacillus* spp. compared to the initial concentration of multi-strain tropical *Bacillus* spp. made a more significant effect on the final results of purification effects, whereas the initial concentration of multi-strain tropical *Bacillus* spp. only affected the purification rate of treatment in the early stage (0–48 h; Figure 3). Reportedly, addition of 10^8^ CFU/ml *B. subtilis* directly to the rearing water could maintain the concentrations of nitrite, ammonia, and nitrate ions within the tolerable ranges for shrimp culture (Zokaeifar et al., 2014); addition of 10^10^ CFU/ml *Bacillus* probiotics in shrimp aquaculture was associated with significantly reduced levels of ammonia, nitrite, and pH in accord with the present study (Nimrat et al., 2012). All of these indicated that our multi-strain tropical *Bacillus* spp. can markedly improve the wastewater quality even at relatively low bacterial concentrations (10^4^ CFU/ml; Figures 3, 4).

COD as an important index is commonly used to measure the total amount of organic matter in water (Li et al., 2018). Reportedly, *Bacillus* as a COD-degrading bacterial consortium can product a variety of digestive enzymes, surfactants, hydrocarbons, phenols, fatty acids, ketones, etc., to accelerate the strong organic matter decomposition as nutrients for its own growth (Arima et al., 1968; Arellano-Carbajal and Olmos-Soto, 2002; Gaur and Tiwari, 2015; Barman et al., 2018; Hura et al., 2018; Li et al., 2018). According to our results (Figures 3A, 4A), multi-strain tropical *Bacillus* spp. as COD-degrading bacterial consortium significantly removed COD in aquaculture wastewater, thereby promoting the virtuous cycle of aquaculture water.

There were many studies on *Bacillus* mineralizing nitrogenous wastes through nitrification and/or denitrification resulting in the reduction of ammonia, nitrate, and nitrite (Nimrat et al., 2012; Barman et al., 2018; Gao et al., 2018; John et al., 2020). Reportedly, ammonia-nitrogen usually displays two forms in water, ionized (NH^4+^), and unionized (NH_3_), which are both toxic to aquatic animals and easily soluble in the cell membrane, thereby being absorbed by gills (John et al., 2020). Nitrate (NO^2–^) converted from ammonia-nitrogen as the end product of nitrification is negligibly toxic compared to ammonia and nitrite. Denitrification can convert nitrate (NO^3–^) into nitrogen gas (N_2_), thereby removing excessive nitrogen from wastewater (Xu et al., 2021). Therefore, we reason that the nitrifying and denitrifying actions of the multi-strain tropical *Bacillus* spp. can in sequence convert ammonia-nitrogen to nitrite, nitrite to nitrate, and then nitrate to nitrogen gas, thereby resulting in the markable declination of ammonia-nitrogen and nitrate in aquaculture wastewater (Figures 3B,C, 4B,C).

Many *Bacillus* spp. are important because of their ability to produce antibiotics/metabolites which have antagonistic effects against pathogenic microorganisms (Sun et al., 2010; Abarike et al., 2018; Kuebutornye et al., 2019). Literatures have proven that aquaculture animals with diet supplemented with *Bacillus* species as probiotics resulted in a better protein efficiency ratio, lower feed conversion ratio, and fast growth (De et al., 2018; Goda et al., 2018; Sadat Hoseini Madani et al., 2018). Nimrat et al. (2012) also reported that forms of *Bacillus* probiotics and modes of probiotic administration *Bacillus* probiotics did not affect the growth and survival of shrimp. In this study, the antagonistic action of the multi-strain tropical *Bacillus* spp. effectively inhibited the pathogenic *Vibrio* spp. to ensure the health of aquatic animals. Meanwhile, in order to be considered as a probiotic, the strains have to be non-toxic to the host. This multi-strain tropical *Bacillus* spp. as a potential biological agent was harmless to shrimp as no mortality was observed.

## Conclusion

In summary, we reported an efficient multi-strain tropical *Bacillus* spp. as a potential probiotic biocontrol agent for large-scale enhancement of mariculture water quality by gradual optimization and large-scale verification. This multi-strain tropical *Bacillus* spp. in the optimum proportion 5: 5: 4 of *B. flexus* QG-3, *B. flexus* NS-4, and *B. licheniformis* XCG-6 as a safe biocontrol agent can not only effectively improve the mariculture water quality but also significantly inhibit *Vibrio* spp. in aquaculture water by COD-degrading, nitrifying, denitrifying, and antagonistic actions. Additionally, the initial *Bacillus* spp. proportion in this multi-strain biocontrol agent as a main factor markedly affected the final purification effects of wastewater, whereas the initial concentration of this multi-strain biocontrol agent only influenced the purification rates instead of purification effects at the 0–48-h period. This work will lay a foundation to develop beneficial microbial agents for wastewater purification and construct an eco-friendly tropical aquaculture model for environmental protection. However, the concept of multi-strain tropical *Bacillus* spp. as a potential probiotic biocontrol agent needs further research to assess the bacterial compositions based on molecular technology, thereby revealing the action mechanisms of the multi-strain *Bacillus* spp. in order to have further understanding of bacterial interactions for application in mariculture systems.

## Data Availability Statement

The original contributions presented in the study are included in the article/Supplementary Material, further inquiries can be directed to the corresponding author/s.

## Author Contributions

WR and ZX designed the study. WR wrote and revised the manuscript. WR, HW, CG, and BX performed the experiments. WR, HL, XZ, XC, and AH analyzed the data. All the authors contributed to the article and approved the submitted version.

## Conflict of Interest

The authors declare that the research was conducted in the absence of any commercial or financial relationships that could be construed as a potential conflict of interest.

## Publisher’s Note

All claims expressed in this article are solely those of the authors and do not necessarily represent those of their affiliated organizations, or those of the publisher, the editors and the reviewers. Any product that may be evaluated in this article, or claim that may be made by its manufacturer, is not guaranteed or endorsed by the publisher.
