# Supplementary material for: Multi-Strain Tropical Bacillus spp. as a Potential Probiotic Biocontrol Agent for Large-Scale Enhancement of Mariculture Water Quality
Source: Front Microbiol. 2021 Aug 11;12:699378. doi: 10.3389/fmicb.2021.699378 (PMC8385719; doi:10.3389/fmicb.2021.699378)
Supplement: Supplementary file 1 [file Data_Sheet_1.docx]

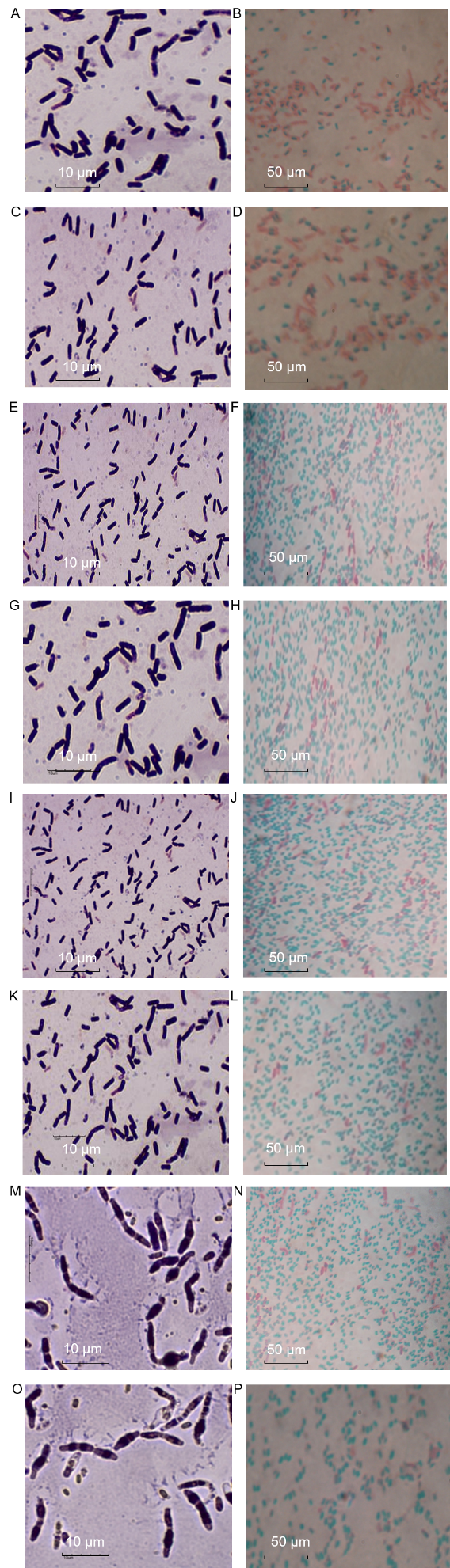


Figure S1 Gram stain and spore stain of *Bacillus licheniformis* XCG-1 (A, B), *Bacillus flexus* NS-2 (C, D), *Bacillus flexus* QG-3 (E, F) and *Bacillus flexus* NS-4 (G, H), *Bacillus licheniformis* XCG-5 (I, J), *Bacillus licheniformis* XCG-6(K, L), *Bacillus flexus* XCG-7 (M, N) and *Bacillus flexus* XCG-8 (O, P).


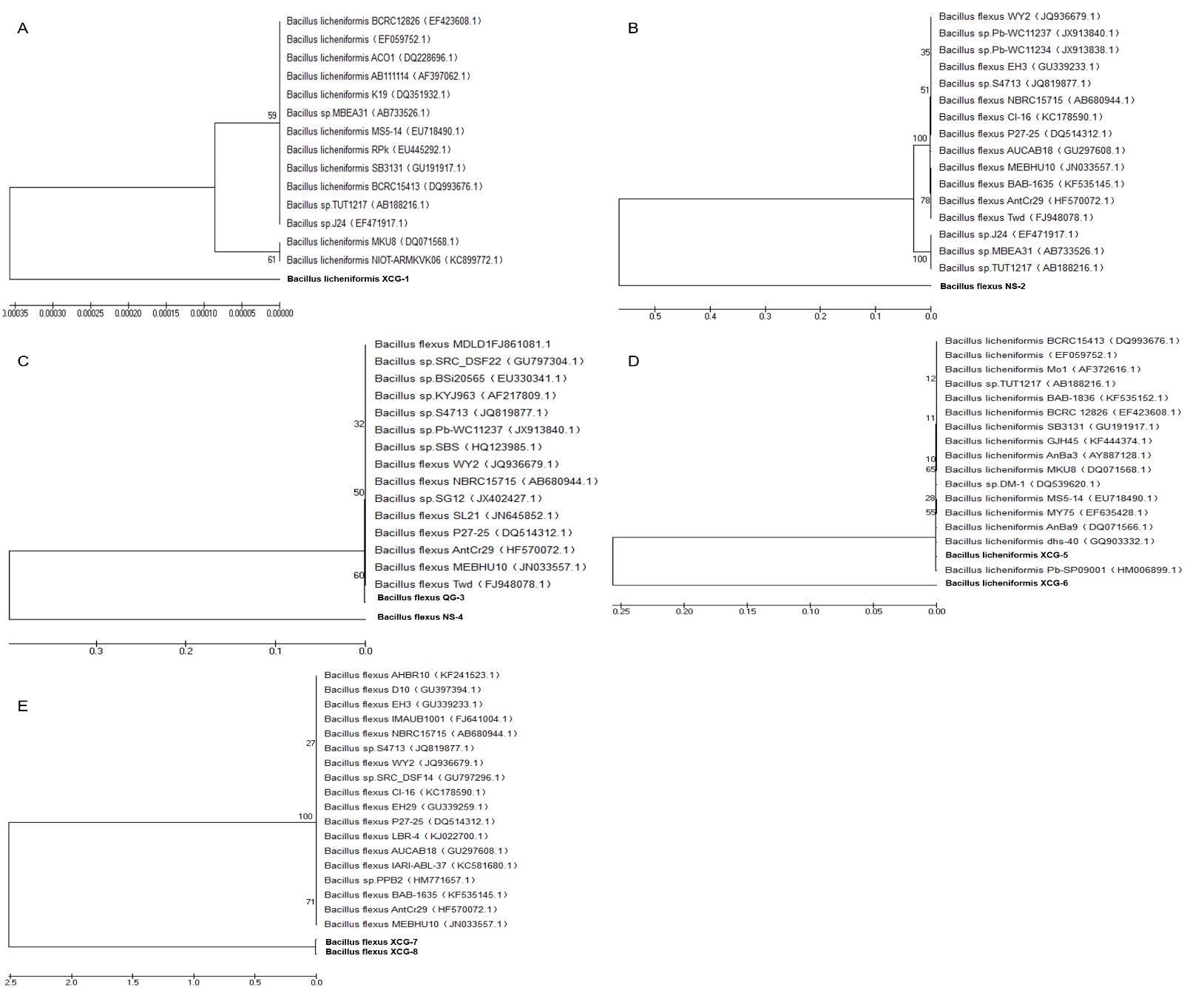


Figure S2 Phylogenetic analyses of *Bacillus licheniformis* XCG-1 (A), *Bacillus flexus* NS-2 (B), *Bacillus flexus* QG-3 and *Bacillus flexus* NS-4 (C), *Bacillus licheniformis* XCG-5 and *Bacillus licheniformis* XCG-6 (D), and *Bacillus flexus* XCG-7 and *Bacillus flexus* XCG-8 (E).

Table S1 COD removal efficiency, activities of Protease and Lipase, and Nitrogen removal rate of target strains.

| *Bacillus licheniformis* XCG-1 | *Bacillus flexus* NS-2 | *Bacillus flexus* QG-3 | *Bacillus flexus* NS-4 | *Bacillus licheniformis* XCG-5 | *Bacillus licheniformis* XCG-6 | *Bacillus flexus* XCG-7 | | *Bacillus flexus* XCG-8 | |
| --- | --- | --- | --- | --- | --- | --- | --- | --- | --- |
| COD removal rate | | Lipase activity（U/mL） | | Protease activity (mm) | | Nitrogen removal rate (%) | | | |
|  |  |  |  |  |  | NH^4+^-N | NO_2_--N | NH^4+^-N | NO^2^--N |
| 82.58±0.79 | 86.66±2.36 | 22.58±0.09 | 18.1±0.19 | 22.38±0.03 | 21.63±0.06 | 89.95±0.56 | 90.56±0.98 | 92.35±1.02 | 91.86±1.53 |


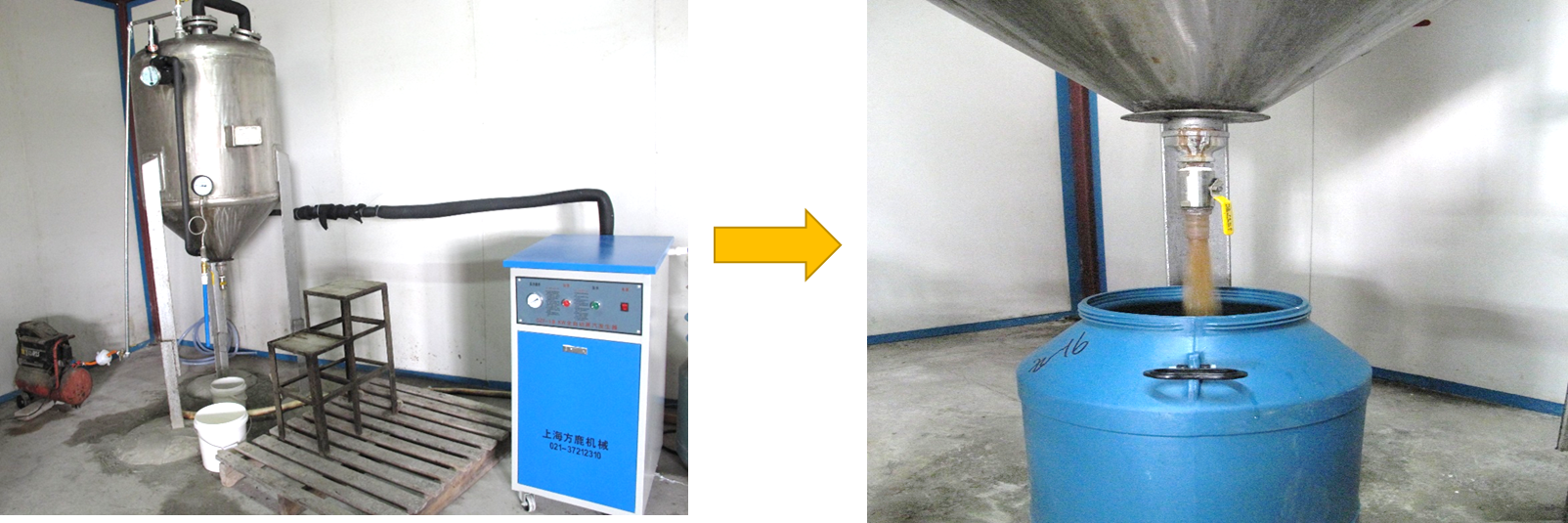


Figure S3 Large-scale production of optimal multi-strain tropical *Bacillus* spp..

Table S2 Physiological biochemical characters of target strains.

| Test | *Bacillus licheniformis* XCG-1 | *Bacillus flexus* QG-3 | *Bacillus flexus* NS-4 | *Bacillus licheniformis* XCG-5 | *Bacillus licheniformis* XCG-6 | Test | *Bacillus flexus* NS-2 | *Bacillus flexus* XCG-7 | *Bacillus flexus* XCG-8 |
| --- | --- | --- | --- | --- | --- | --- | --- | --- | --- |
| Voges-Proskaur (V-P) | + | + | + | + | + | 8%NaCl | + | + | + |
| Propionate | + | + | + | + | + | 15%NaCl | + | + | + |
| Amylolysis | + | + | + | + | + | 50℃ | + | + | + |
| Gelatin liquefaction | + | + | + | + | + | 60℃ | + | + | + |
| pH>7 after V-P culture | - | - | - | - | - | Catalase | + | + | + |
| Citrate | + | + | + | + | + | Casein hydrolysate | + | + | + |
| 55℃ | + | + | + | + | + | Amylase | + | + | + |
|  |  |  |  |  |  | Acid from |  |  |  |
| pH5.7 | + | + | + | + | + | D-fructose | + | + | + |
| pH6.8 | + | + | + | + | + | D-glucose | + | + | + |
| NaCl 5% | + | + | + | + | + | Sucrose | + | + | + |
| NaCl 7% | + | + | + | + | + | D-lactose | - | - | + |

Note: + = positive reaction, - = negative reaction.

Figure S4 The purification effect of multi-strain tropical *Bacillus* spp. on DO.
